# Photodynamic Action of Hypocrellin A and Hypocrellin B against Cancer—A Review

**DOI:** 10.3390/ph18121847

**Published:** 2025-12-03

**Authors:** Jinju Huang, Siu Kan Law, Albert Wing Nang Leung, Chuanshan Xu

**Affiliations:** 1 Intensive Care Unit Ward 1, The Affiliated Panyu Central Hospital, Guangzhou Medical University, Guangzhou 511400, China; orangesking@163.com; 2 Independent Researcher, Hong Kong SAR, China; siukanlaw@hotmail.com; 3 School of Graduate Studies, Lingnan University, Tuen Mun, Hong Kong SAR, China; albertleung@ln.edu.hk; 4 Guangzhou Municipal and Guangdong Provincial Key Laboratory of Molecular Target & Clinical Pharmacology, The NMPA and State Key Laboratory of Respiratory Disease, School of Pharmaceutical Sciences, Guangzhou Medical University, Guangzhou 511436, China

**Keywords:** photodynamic therapy, hypocrellin A, hypocrellin B, cancer

## Abstract

Cancer is a major global health concern, affecting nearly 20 million individuals annually, according to the International Agency for Research on Cancer (IARC). There are some unconventional and conventional treatments for cancer. Typically, they span a wide spectrum of conventional and advanced therapeutic approaches, such as photodynamic therapy (PDT). This has long been valued for its non-invasive, targeted, and minimally toxic approach in the management of cancer. More importantly, PDT results in fewer operative and post-operative major complications, faster recovery times, reduced operating time, and saved costs. There are two types of photosensitizers in PDT, including synthetics (e.g., hematoporphyrin derivative, photofrin II, verteporfin) and natural (e.g., Hypocrellin A (HA) and Hypocrellin B (HB)). Nine electronic databases—WanFang Data, PubMed, ScienceDirect, Scopus, Web of Science, Springer Link, SciFinder, and the China National Knowledge Infrastructure (CNKI)—were systematically searched for this review, covering the literature published within the past 20 to 30 years (time range), without language restrictions. Studies were included if they were identified using the keywords Hypocrellin A, Hypocrellin B, photodynamic therapy, and cancer (inclusion criteria). All eligible papers were collected, critically analyzed, and summarized. Duplicate records were excluded during the screening process (exclusion criteria). HA and HB, derived from the fungus *Hypocrella bambusae*, offer a natural alternative with lower toxicity. However, these compounds are still in the in vitro or in vivo, and must meet rigorous standards for “quality”, “safety”, “efficacy”, “pharmacokinetics”, as well as “regulatory compliance” before entering clinical trials. “Curcumin” is a successful PS for traditional Chinese medicine used in PDT during clinical study and it is used as a benchmark for HB. Currently, scientists are paying attention to “nanotechnology” that enhances hypocrellin’s properties in PDT for achieving clinical goals, but further investigations are required.

## 1. Introduction

Cancer is a disease caused by uncontrolled cell proliferation and transformed cells are subject to evolution by natural selection [1]. There are nearly 20 million people who have cancer every year, estimated by the International Agency for Research on Cancer (IARC) [2]. Lung, breast, prostate, stomach, and liver cancers are the most common [3].

Generally, there are some unconventional and conventional treatments containing radiotherapy, surgery, chemotherapy, and advanced technologies like gene therapy, stem cell therapy, natural antioxidants, targeted therapy, photodynamic therapy, nanomedicine, and precision medicine which are available to diagnose and treat cancer [4].

“Invasive treatment” such as surgery may cause infections, complications from anesthesia, and a longer recovery period. More importantly, it can increase the risk of tumor recurrence after surgery and high costs [5]. “Non-invasive treatment” like photodynamic therapy (PDT) result in fewer operative and post-operative major complications, faster recovery times, less damage to the immune system, even reduced operating time, and saved costs [6]. The advantages of using PDT in cancer treatment are attributed to its selective killing of cancer cells, without damaging the living cells or tissues [7].

Cancer therapy has undergone significant evolution over the past century, transitioning from broadly cytotoxic treatments to more targeted and minimally invasive modalities. Because of the PDT benefit, it has emerged as a clinically viable approach, integrating light activation with photosensitizing agents to induce localized cytotoxicity. Its mechanistic foundations trace back to the early 20th century and gained substantial medical traction from the 1970s to the 1980s [8]. A landmark clinical application occurred in 1976, when hematoporphyrin was first used as a photosensitizer (PS) in PDT for bladder cancer [9]. This has been continuously applied to a variety of malignancies using PSs tailored to tumor type and anatomical site—hematoporphyrin derivatives for breast and lung cancers [10,11], Photofrin II for pleural and gastric cancers [12,13], 5-aminolevulinic acid (ALA) for superficial basal cell carcinoma [14], methyl aminolevulinate (MAL) for non-melanoma skin cancers [15], verteporfin for pancreatic cancer [16], etc.

They are synthetic PSs and have some limitations in clinical usage. Hematoporphyrin possesses poor light penetration depth, high photosensitivity leading to a long period of light avoidance after treatment, and a lack of selectivity for tumor sites, which can cause systemic side effects [17]; Photofrin II has a complex composition and a low light absorption rate [18]; ALA has limited depth of tissue penetration, causes significant pain during treatment, and requires strict light avoidance protocols after the procedure [19]; MAL is associated with pain, variable long-term efficacy, and specific application constraints [20]. Meanwhile, there are still concerns regarding its phototoxicity, cost, and the long-term biocompatibility of synthetic PSs which has catalyzed interest in natural alternatives.

Natural photosensitizers—derived from botanical, microbial, or marine sources—offer promising advantages in terms of safety, accessibility, and ecological sustainability, which are increasingly being explored for their potential to enhance PDT efficacy and minimize adverse effects, like protoporphyrin IX, chlorophylls, flavonoids, anthocyanins, carotenoids, and curcumin. Protoporphyrin IX uses artificial daylight to improve skin antisepsis for orthopedic surgeries [21]; chlorophylls have better selectivity of tumor tissue destruction and lack severe local and systemic complications [22]; flavonoids as adjuvant agents to enhance therapeutic efficacy and reduce side effects [23]; anthocyanins enhance cell death when combined with light, dark cytotoxicity, and protect cells from damage, reduce inflammation, as well as possess antimicrobial properties [24]; carotenoids can quench singlet oxygen and free radicals for helping and protecting normal cells from oxidative damage and inflammation [25]; curcumin overcome drug resistance by modulating apoptosis pathways, it has potential for deeper tissue penetration when used in nanoformulations [26]. There are other natural PSs such as Hypocrellin A (HA) and Hypocrellin B (HB).

Unlike previous reviews studied, that only broadly surveyed PDT agents, this article focuses specifically on HA and HB as natural PSs in cancer phototherapy, which provides a comprehensive overview of their chemical characteristics, mechanisms of action, and photophysical properties, alongside a discussion of their application in traditional and nanotechnology-enhanced PDT platforms. The article further discusses and evaluates the preclinical or clinical investigations that underscore their therapeutic promise in oncological contexts.

### 1.1. Photosensitizers from Natural Herbal Compounds

**Figure 1:**
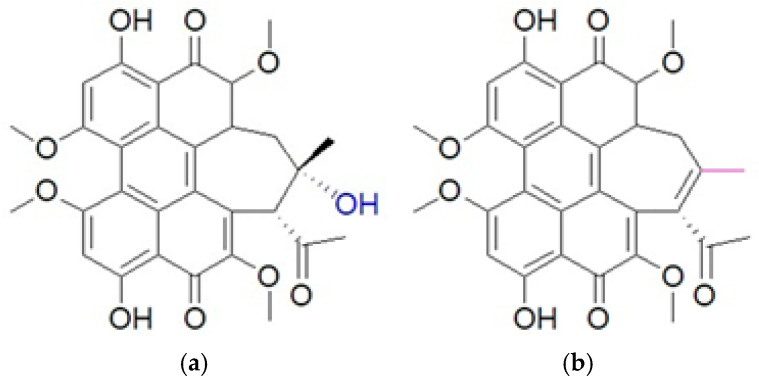
Chemical structure of (**a**) Hypocrellin A (HA) and (**b**) Hypocrellin B (HB).

Hypocrellins are natural perylenequinone pigments derived from the parasitic fungus *Hypocrella bambusae* [27], which were used in traditional Chinese medicine with a long history, such as anticancer and antiviral functions [28]. It is classified as Hypocrellin A (HA) and Hypocrellin B (HB) (Figure 1), containing different side chain structures with photochemical properties in the application of PDT. HA has a five-membered ring containing a hydroxyl group, whereas HB contains a six-membered ring with a methoxy group. The structures are differences that lead to changes in their ability for their photochemical reactions as a PS in PDT [29]. HA is more polar than HB because of an existing hydroxyl group. The oxygen atom from the hydroxyl group becomes electronegative and draws electron density, producing a dipole within the chemical structure [30].

### 1.2. Photodynamic Therapy (PDT)

The general principle of PDT is light activation with a specific wavelength on the PS that reacts with the oxygen to produce reactive oxygen species (ROS) and eradicates the infection or tumors [31]. It depends on the three major components in determining PDT efficacy: light source, PS, and molecular oxygen [32]. The choice of light source relates to the target location, usage of PS, and the dose (energy) or frequency of light to be applied [33]. PS, like HA and HB, are substances that become activated by a specific wavelength of light to produce ROS to destroy the target cells [34]. ROS are produced by the PS transfer energy process, creating a toxic and hypoxic environment in the tumors [35].

Typically, there are two main photochemical mechanisms, including type I radical-mediated and type II ^1^O_2_ reactions. Type I reaction is the light source that excites the triplet state of PS, and interacts with biomolecules or molecular oxygen through the transfer of an electron or hydrogen, forming a highly ROS such as superoxide anion (O_2_^•−^), hydroxyl radicals (•OH) and hydrogen peroxide (H_2_O_2_) [36]. Type II reaction is the light source that excites PS and transfers its energy directly to O_2_, converting it into ^1^O_2_, and other ROS. Type II is dominant in an oxygen-rich environment, and Type I is dominant in a hypoxic environment [37].

**Figure 2:**
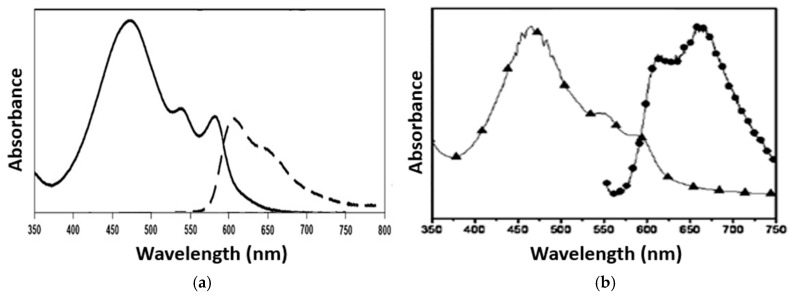
Absorption (solid line) and fluorescence (dashed line) of (**a**) Hypocrellin A (HA); absorption (triangle with line) and fluorescence (spot with line) of (**b**) Hypocrellin B (HB) in DMSO. Copied from references [40,41].

Hypocrellins may undergo these two types of mechanisms. HB is a more efficient PS compared to HA during the PDT process, because it exhibits a higher potential to generate ROS in an oxygen-rich environment, and can generate its semiquinone radical anion (HB•^−^) under a hypoxic environment. Meanwhile, the generation of ROS from HB is much stronger than HA, which indicates it is a more phototherapeutic agent for killing the target cells in PDT. However, HA and HB have similar spectroscopic and photophysical behaviors, they are highly sensitive to the solvent medium and molecular interaction [38]. The absorption and emission spectra of HA and HB reveal distinct photophysical properties. A primary activation wavelength of HA is around 465 nm, while HB shows activation between 460 and 470 nm in the blue region of the visible spectrum. HB exhibits more in the red region—a shifted absorption band compared to HA, with a dominant band at 540 to 560 nm, and a secondary peak at 465 nm (Figure 2). In contrast, HA can be efficiently activated by yellow light at 585 nm or red light at 630 nm. Fluorescence emission for HA and HB lies in the red region, HA peaks near 640 nm, and HB shows an emission maximum at 645 nm within the 630 to 660 nm band [39]. These spectral characteristics underpin their application in cancer therapy.

## 2. Traditional Strategy of HB-PDT for Cancer

HB acts as a PS in PDT under the specific wavelength of light, usually in the region of blue to red light from 470 to 700 nm, producing the effective ROS to induce apoptosis in different types of cancers, such as skin, lung, ovarian, and breast cancers (Table 1). The common apoptosis in PDT is the genetically regulated form of cell death that consists of intrinsic and extrinsic pathways [42]. The intrinsic pathway is initiated by internal cellular stress, leading to permeabilization or damage of the mitochondrial outer membrane. This process is regulated by the BCL-2 family of proteins, where the downregulation of anti-apoptotic members (e.g., Bcl-2, Bcl-xL) and upregulation of pro-apoptotic members (e.g., Bax, Bak) facilitate the release of cytochrome c, while the extrinsic pathway is triggered by the binding of death ligands (e.g., TNF-α) to death receptors on the cell membrane. This interaction recruits adaptor proteins forming the death-inducing signaling complex, which activates caspase-8, then subsequently activates caspase-3, executing the apoptotic process [43]. HB demonstrates dual photodynamic pathways, producing both singlet oxygen and hydroxyl radicals under 470 to 700 nm irradiation in the mechanisms discussed in 1.2. Its mitochondrial localization and ROS diversity suggest enhanced efficacy in hypoxic conditions, as supported by its mechanistic profile in Table 1.

**Table 1:** Examples of the traditional strategy of HB-PDT for cancer.

	Study	Experiment Parameters	Consequence	References
1	Effects of photodynamic therapy using Red LED-light combined with hypocrellin B on apoptotic signaling in cutaneous squamous cell carcinoma A431 cells	Absorption (λ_abs_): 540 to 560 nm (dominant band), secondary peak at 465 nm. Absorption maximum (λ_abs max_): 550 nm for molar extinction coefficients (ε): 4.2 × 10^4^ M^−1^·cm^−1^ Emission (λ_em_): 630 to 660 nm with λ_em max_: 645 nm Singlet oxygen quantum yields (ΦΔ): 0.54 Cytotoxicity IC_50_: 0.45 μM in A431 cells	HB-PDT induced apoptosis in A431 cells through a mitochondria-mediated apoptotic pathway and was possible in the treatment of cutaneous squamous cell carcinoma.	[44]
2	Effect of photodynamic therapy with hypocrellin B on apoptosis, adhesion, and migration of cancer cells	Absorption (λ_abs_): 540 to 560 nm (dominant band), secondary peak at 465 nm. Absorption maximum (λ_abs max_): 550 nm for molar extinction coefficients (ε): 4.0 × 10^4^ M^−1^·cm^−1^ Emission (λ_em_): 630 to 660 nm with λ_em max_: 645 nm Singlet oxygen quantum yields (ΦΔ): 0.52 Cytotoxicity IC_50_: ~0.50 μM in HeLa, and A431 cells	HB-PDT induced apoptosis and inhibited adhesion and migration of ovarian cancer cells in vitro.	[45]
3	Evaluation of hypocrellin B in a human bladder tumor model in experimental photodynamic therapy: biodistribution, light dose and drug-light interval effects	Absorption (λ_abs_): 540 to 560 nm (dominant band), secondary peak at 465 nm. Absorption maximum (λ_abs max_): 550 nm for molar extinction coefficients (ε): 4.1 × 10^4^ M^−1^·cm^−1^ Emission (λ_em_): 630 to 660 nm with λ_em max_: 645 nm Singlet oxygen quantum yields (ΦΔ): 0.53 Cytotoxicity IC_50_: 0.4 to 0.6 μM in MGH cells	HB-PDT contributed to the effect of vascular damage on the tumor, leading to destruction.	[46]
4	In vitro and in vivo antitumor activity of a novel hypocrellin B derivative for photodynamic therapy	Absorption (λ_abs_): 540 to 560 nm (dominant band), secondary peak at 465 nm. Absorption maximum (λ_abs max_): 550 nm for molar extinction coefficients (ε): ~4.3 × 10^4^ M^−1^·cm^−1^ Emission (λ_em_): 630 to 660 nm with λ_em max_: 645 nm Singlet oxygen quantum yields (ΦΔ): ~0.56 Cytotoxicity IC_50_: ~0.42 μM in HeLa cells In vivo pharmacokinetics: xenograft models	HB with Schiff-base-PDT induced the potential of mitochondrial inner membrane, cytochrome c release, caspase-3 activation, and subsequent apoptotic death for cancer.	[47]
5	Effects of photodynamic therapy using yellow LED-light with concomitant hypocrellin B on apoptotic signaling in keloid fibroblasts	Absorption (λ_abs_): 540 to 560 nm (dominant band), secondary peak at 465 nm. Absorption maximum (λ_abs max_): 550 nm for molar extinction coefficients (ε): ~3.8 × 10^4^ M^−1^·cm^−1^ Emission (λ_em_): 570 to 590 nm with λ_em max_: 580 nm Singlet oxygen quantum yields (ΦΔ): ~0.51 Cytotoxicity IC_50_: ~0.6 μM in keloid fibroblast In vivo pharmacokinetics: xenograft models	HB-PDT induced BAX upregulation and BCL-2 downregulation in KFB cells, leading to the elevation of intracellular free Ca^2+^ and activation of caspase-3 in the keloid fibroblasts.	[48]
6	Apoptosis of breast cancer cells induced by hypocrellin B under light-emitting diode irradiation	Absorption (λ_abs_): 540 to 560 nm (dominant band), secondary peak at 465 nm. Absorption maximum (λ_abs max_): 550 nm for molar extinction coefficients (ε): ~4.2 × 10^4^ M^−1^·cm^−1^ Emission (λ_em_): 630 to 660 nm with λ_em max_: 645 nm Singlet oxygen quantum yields (ΦΔ): ~0.52 Cytotoxicity IC_50_: ~0.45 μM in MDA-MB-231 cells	HB-PDT exhibited a dose-dependent manner and induced apoptotic cell death in breast cancer.	[49]
7	A glutathione responsive photosensitizer based on hypocrellin B for photodynamic therapy	Absorption (λ_abs_): 540 to 560 nm (dominant band), secondary peak at 465 nm. Absorption maximum (λ_abs max_): 550 nm for molar extinction coefficients (ε): ~4.5 × 10^4^ M^−1^·cm^−1^ Emission (λ_em_): 590 to 610 nm with λ_em max_: 600 nm Singlet oxygen quantum yields (ΦΔ): ~0.58 Cytotoxicity IC_50_: ~0.38 μM in HeLa cells In vivo pharmacokinetics: GSH-triggered activation	HB-PDT was activated by glutathione to induce cancer cells to achieve recuperative fluorescence and singlet oxygen generation.	[50]
8	Involvement of the Mitochondria-Caspase Pathway in HeLa Cell Death Induced by 2-Ethanolamino-2-Demethoxy-17-Ethanolimino-Hypocrellin B (EAHB)-Mediated Photodynamic Therapy	Absorption (λ_abs_): 540 to 560 nm (dominant band), secondary peak at 465 nm. Absorption maximum (λ_abs max_): 550 nm for molar extinction coefficients (ε): ~4.3 × 10^4^ M^−1^·cm^−1^ Emission (λ_em_): 630 to 660 nm with λ_em max_: 645 nm Singlet oxygen quantum yields (ΦΔ): ~0.55 Cytotoxicity IC_50_: ~0.41 μM in HeLa cells	2-ethanolamino-2-demethoxy-17-ethanolimino-HB-PDT induced a cytochrome c release from the mitochondria into the cytosol, followed by the activation of caspase 3 and caspase 9 in HeLa cells.	[51]
9	Biophysical evaluation of two red-shifted hypocrellin B derivatives as novel PDT agents	Absorption (λ_abs_): 540 to 560 nm (dominant band), secondary peak at 465 nm. Absorption maximum (λ_abs max_): 550 nm for molar extinction coefficients (ε): ~4.6 to 4.8 × 10^4^ M^−1^·cm^−1^ Emission (λ_em_): 630 to 660 nm with λ_em max_: 645 nm Singlet oxygen quantum yields (ΦΔ): ~0.55 to 0.60 Cytotoxicity IC_50_: ~0.35 to 0.42 μM in BGC-823 cells In vivo pharmacokinetics: decrease dark toxicity with longer circulation	HB derivatives-PDT enhanced the singlet oxygen generating efficiency and increased light-dependent cytotoxicity on colon cancer.	[52]
10	A novel hypocrellin B derivative designed and synthesized by taking consideration to both drug delivery and biological photodynamic activity	Absorption (λ_abs_): 540 to 560 nm (dominant band), secondary peak at 465 nm. Absorption maximum (λ_abs max_): 550 nm for molar extinction coefficients (ε): ~4.2 × 10^4^ M^−1^·cm^−1^ Emission (λ_em_): 630 to 660 nm with λ_em max_: 645 nm Singlet oxygen quantum yields (ΦΔ): ~0.54 Cytotoxicity IC_50_: ~0.47 μM in endothelial cells In vivo pharmacokinetics: improve solubility and bioavailability	17-3-amino-1-propane-sulfonic acid-HB Schiff-base-PDT delivered into target tissues to the solid tumor via blood circulation after intravenous injection.	[53]
11	Exploitation of immune response-eliciting properties of hypocrellin photosensitizer SL052-based photodynamic therapy for eradication of malignant tumors	Absorption (λ_abs_): 540 to 560 nm (dominant band), secondary peak at 465 nm. Absorption maximum (λ_abs max_): 550 nm for molar extinction coefficients (ε): ~4.4 × 10^4^ M^−1^·cm^−1^ Emission (λ_em_): 630 to 660 nm with λ_em max_: 645 nm Singlet oxygen quantum yields (ΦΔ): ~0.55 Cytotoxicity IC_50_: ~0.40 to 0.50 μM in endothelial cells In vivo pharmacokinetics: enhance immune activation	HB diaminophenyl derivative-PDT indicated a further increase in the number of cells in tumor-draining lymph nodes and in degranulating CD8+ cells, and the amplification of the immune response induced by PDT.	[54]

## 3. Nanotechnology of HB-PDT for Cancer

HB has some disadvantages as a PS, including low water solubility and weak absorption in the phototherapeutic window which pose challenges to its use in treating solid tumors (Table 2) [55]. Poor water solubility of PS may cause aggregation, especially for the HB with a methoxy group; this is hydrophobic and interacts by the π-π stacking to influence the generation of ROS on PDT therapeutic effectiveness.

Nanotechnology is a suitable method to address these problems. It develops the drug delivery systems to enhance the therapeutic efficacy of PDT, such as the solubility and stability of PS. This contains a series of nanoparticle platforms, such as micelles, liposomes, graphene oxides, and polymeric nanoparticles [56]. One of the specific processes is usually used in drug delivery systems, “Nanonization”, which is the reduction in particle loading HB to the nanoscale, increasing the surface area and consequently the saturation solubility and dissolution rate, and resulting in higher bioavailability [57].

Micelles are nanoscale spherical assemblies formed from amphiphilic polymers such as PEG-PLA or Pluronic F127, comprising a hydrophobic core and hydrophilic shell [58]. HB is a hydrophobic PS, which can be effectively encapsulated within the micellar core, protecting it from premature degradation, and enhancing its dispersion in biological fluids. This formulation significantly improves HB’s aqueous solubility, enabling intravenous administration and prolonged systemic circulation. Micellar HB demonstrates superior tumor accumulation and cellular uptake compared to its free form, primarily via endocytosis. The intracellular concentration of micellar HB is markedly higher, and its red-shifted absorption profile facilitates deeper tissue light penetration, further enhancing its photodynamic efficacy.

Liposomes are spherical vesicles composed of phospholipid bilayers surrounding an aqueous core [59]. HB is a hydrophobic PS embedded within the lipid bilayer. Its mechanism to improve solubility, tumor accumulation, and light penetration is similar to micelles.

Graphene oxide is a two-dimensional carbon-based nanomaterial with abundant oxygen-containing functional groups that are electron-donating groups like hydroxyl, carboxyl, and epoxy [60]. HB molecules are adsorbed on or covalently conjugated to the graphene oxide via π-π stacking, hydrogen bonding, or amide linkage.

Polymeric nanoparticles are typically made from biodegradable polymers such as poly (lactic-co-glycolic acid) (PLGA) or chitosan [61]. HB is loaded into the polymer matrix via nanoprecipitation, emulsion, or self-assembly techniques.

The mechanisms by which liposomes, graphene oxide, and polymeric nanoparticles improve HB’s solubility, tumor targeting, and light-mediated activation are fundamentally similar to those of micelles, yet each platform offers distinct advantages in terms of pharmacokinetics, subcellular localization, and combinatorial therapeutic potential.

**Table 2:** Examples of the nanotechnology of HB-PDT for cancer.

	Study	Experiment Parameters	Consequence	References
1	Hypocrellin B and paclitaxel-encapsulated hyaluronic acid-ceramide nanoparticles for targeted photodynamic therapy in lung cancer	Absorption (λ_abs_): 540 to 560 nm (dominant band), secondary peak at 465 nm. Absorption maximum (λ_abs max_): 550 nm for molar extinction coefficients (ε): ~4.3 × 10^4^ M^−1^·cm^−1^ Emission (λ_em_): 630 to 660 nm with λ_em max_: 645 nm Singlet oxygen quantum yields (ΦΔ): ~0.56 Cytotoxicity IC_50_: ~0.42 μM in A549 cells In vivo pharmacokinetics: reduce systemic toxicity	HB and paclitaxel-encapsulated hyaluronic acid-ceramide nanoparticles-PDT increased the therapeutic efficacy on lung cancer in mice, because of the overexpression of low-density lipoprotein receptors.	[62]
2	Hypocrellin B-loaded, folate-conjugated polymeric micelle for intraperitoneal targeting of ovarian cancer in vitro and in vivo	Absorption (λ_abs_): 540 to 560 nm (dominant band), secondary peak at 465 nm. Absorption maximum (λ_abs max_): 550 nm for molar extinction coefficients (ε): ~4.2 × 10^4^ M^−1^·cm^−1^ Emission (λ_em_): 630 to 660 nm with λ_em max_: 645 nm Singlet oxygen quantum yields (ΦΔ): ~0.53 Cytotoxicity IC_50_: ~0.46 μM in SKOV3 ovarian cancer cells In vivo pharmacokinetics: reduce systemic toxicity	HB/FA-PEG-PLA micelles possessed a high drug-loading capacity, good biocompatibility, controlled drug release, and enhanced targeting, as well as the antitumor effect of PDT on ovarian cancer.	[63]
3	Liposomal hypocrellin B as a potential photosensitizer for age-related macular degeneration: pharmacokinetics, photodynamic efficacy, and skin phototoxicity in vivo	Absorption (λ_abs_): 540 to 560 nm (dominant band), secondary peak at 465 nm. Absorption maximum (λ_abs max_): 550 nm for molar extinction coefficients (ε): ~4.2 × 10^4^ M^−1^·cm^−1^ Emission (λ_em_): 630 to 660 nm with λ_em max_: 645 nm Singlet oxygen quantum yields (ΦΔ): ~0.52 Cytotoxicity IC_50_: ~0.48 μM in ARPE-19 retinal pigment epithelial cells In vivo pharmacokinetics: selective accumulation in ocular tissues	Liposomal HB was an effective photosensitizer for vascular-targeted PDT of age-related macular degeneration.	[64]
4	High-efficiency loading of hypocrellin B on graphene oxide for photodynamic therapy	Absorption (λ_abs_): 540 to 560 nm (dominant band), secondary peak at 465 nm. Absorption maximum (λ_abs max_): 550 nm for molar extinction coefficients (ε): ~4.1 × 10^4^ M^−1^·cm^−1^ Emission (λ_em_): 630 to 660 nm with λ_em max_: 645 nm Singlet oxygen quantum yields (ΦΔ): ~0.54 Cytotoxicity IC_50_: ~0.43 μM in HeLa cells	HB was loaded on the graphene oxide, resulting in efficient generation of singlet oxygen during the PDT process, which was actively taken up into the cytosol of tumor cells.	[65]
5	Biodegradable Hypocrellin B nanoparticles coated with neutrophil membranes for hepatocellular carcinoma photodynamics therapy effectively via JUNB/ROS signaling	Absorption (λ_abs_): 540 to 560 nm (dominant band), secondary peak at 465 nm. Absorption maximum (λ_abs max_): 550 nm for molar extinction coefficients (ε): ~4.3 × 10^4^ M^−1^·cm^−1^ Emission (λ_em_): 630 to 660 nm with λ_em max_: 645 nm Singlet oxygen quantum yields (ΦΔ): ~0.56 Cytotoxicity IC_50_: ~0.41 μM in HepG2 cells In vivo pharmacokinetics: reduce systemic toxicity	The neutrophil membrane-coated HB nanoparticles significantly increased the therapeutic efficacy of PDT to suppress the growth of hepatocellular carcinoma, because of reactive oxygen species production and mitochondrial dysfunction via the inhibition of JunB proto-oncogene expression.	[66]
6	Hypocrellin B-encapsulated nanoparticle-mediated rev-caspase-3 gene transfection and photodynamic therapy on tumor cells	Absorption (λ_abs_): 540 to 560 nm (dominant band), secondary peak at 465 nm. Absorption maximum (λ_abs max_): 550 nm for molar extinction coefficients (ε): ~2.5 to 3.5 × 10^4^ M^−1^·cm^−1^ Emission (λ_em_): 630 to 660 nm with λ_em max_: 645 nm Singlet oxygen quantum yields (ΦΔ): ~0.52 to 0.65 Cytotoxicity IC_50_: ~0.5 to 1.2 μM in nanoparticle formulation	HB-encapsulated nanoparticle was an efficient gene carrier and a novel photosensitizer in PDT for enhancing the transfection efficiency of rev-caspase-3 gene in the nasopharyngeal carcinoma.	[67]
7	Hypocrellin B doped and pH-responsive silica nanoparticles for photodynamic therapy	Absorption (λ_abs_): 540 to 560 nm (dominant band), secondary peak at 465 nm. Absorption maximum (λ_abs max_): 550 nm for molar extinction coefficients (ε): ~3.2 × 10^4^ M^−1^·cm^−1^ Emission (λ_em_): 630 to 660 nm with λ_em max_: 645 nm Singlet oxygen quantum yields (ΦΔ): ~0.58 Cytotoxicity IC_50_: ~0.8 to 1.5 μM in HeLa, and HepG2 cells	HB-doped silica nanoparticles were effective in killing tumor cells by PDT, which regulated the singlet oxygen generation efficiency through the “inner filter” effect.	[68]
8	Biodegradable hypocrellin derivative nanovesicle as a near-infrared light-driven theranostic for dually photoactive cancer imaging and therapy	Absorption (λ_abs_): 540 to 560 nm (dominant band), secondary peak at 465 nm. Absorption maximum (λ_abs max_): 550 nm for molar extinction coefficients (ε): ~4.1 × 10^4^ M^−1^·cm^−1^ Emission (λ_em_): 630 to 660 nm with λ_em max_: 645 nm Singlet oxygen quantum yields (ΦΔ): ~0.62 Cytotoxicity IC_50_: ~0.6 to 1.1 μM in HeLa, and MCF-7 cells	Amino-substituted HB derivative contained 1,2-diamino-2-methyl propane, possessed high photothermal stability, enhanced tumor accumulation, and a suitable biodegradation rate, as well as high generation of singlet oxygen during the PDT process for cancer therapy.	[69]
9	Hypocrellin derivative-loaded calcium phosphate nanorods as NIR light-triggered phototheranostic agents with enhanced tumor accumulation for cancer therapy	Absorption (λ_abs_): 540 to 560 nm (dominant band), secondary peak at 465 nm. Absorption maximum (λ_abs max_): 550 nm for molar extinction coefficients (ε): ~4.3 × 10^4^ M^−1^·cm^−1^ Emission (λ_em_): 630 to 660 nm with λ_em max_: 645 nm Singlet oxygen quantum yields (ΦΔ): ~0.60 Cytotoxicity IC_50_: ~0.5 to 1.0 μM in tumor accumulation	HB derivative-loaded calcium phosphate nanorods improved the singlet oxygen generation and enhanced cellular uptake efficiency in vitro and in vivo, offering potentially promising fluorescence imaging-guided photodynamic therapy of cancer for clinical applications.	[70]
10	Comparative study of free and encapsulated hypocrellin B on photophysical-chemical properties, cellular uptake, subcellular distribution, and phototoxicity	Absorption (λ_abs_): 540 to 560 nm (dominant band), secondary peak at 465 nm. Absorption maximum (λ_abs max_): 550 nm for molar extinction coefficients (ε): ~2.8 to 3.2 × 10^4^ M^−1^·cm^−1^ Emission (λ_em_): 630 to 660 nm with λ_em max_: 645 nm Singlet oxygen quantum yields (ΦΔ): ~0.58 to 0.65 Cytotoxicity IC_50_: ~0.6 to 1.2 μM in HepG2 cells	Hydrophobic HB was encapsulated into liposomes or poly (lactic-co-glycolic acid) nanoparticles induced pronounced phototoxicity with substantial reactive oxygen species production, confirming the robust PDT effect on cancer.	[71]

## 4. Traditional Strategy of HA-PDT for Cancer

Similarly, HA also acts as a PS in PDT under the specific wavelength of light, but it is usually in the region of red light from 565 to 610 nm, producing the effective ROS to induce apoptosis in different types of cancers, such as skin and lung cancers (Table 3). The common apoptosis in PDT consists of intrinsic and extrinsic pathways relating to the permeabilization or damage of the mitochondrial outer membrane.

## 5. Nanotechnology of HA-PDT for Cancer

The reasons for using nanotechnology are the same as HB (Table 4).

## 6. Discussion

PDT was approved by the U.S. Food and Drug Administration (FDA) over forty years ago and has since gained recognition as a valuable adjuvant treatment for solid tumors [7]. Clinical evidence increasingly supports its ability to target residual microscopic disease, particularly through interstitial light delivery that penetrates directly into tumor masses. This approach enables PDT to treat large, deeply embedded tumors that would otherwise require extensive surgical resection, as highlighted by Hopper [79]. Despite these advances, no clinical trials have yet been published on the use of HB-PDT or HA-PDT in cancer therapy. Current investigations remain in the experimental or preclinical phase, with early-stage results summarized in Table 1, Table 2, Table 3 and Table 4.

The photodynamic action of HA and HB against cancer still has some considerations at present including: (1) Why use HA or HB as a PS? (2) Which one is more suitable for PDT? (3) What are the limitations and improvements of using these PS? (4) Compared to other Chinese medicine PSs, are there any benefits? (5) How do these PS in PDT apply to a clinical study? These doubts are valuable to consider.

HA and HB are natural products that are derived from the parasitic fungus *Hypocrella bambusae*. They exhibit lower toxicity compared to hematoporphyrin derivatives like photofrin II [80]. It is safer and reliable, which should not be toxic in the absence of light (dark toxicity). Meanwhile, HA and HB exhibit strong absorption peaks primarily in the visible blue-green region from 460 to 500 nm. It has moderate absorption extending into the red region from 600 to 650 nm) only. The red-light absorption is limited compared to second-generation photosensitizers. This partial overlap still offers some potential for tissue penetration and solid tumor treatment, especially in the nanocarrier-based delivery systems or structural modification.

HB is more suitable as a PS in PDT than HA, because it has a high efficiency in the intersystem crossing to excite the singlet to triplet state for generating the ^1^O_2_ and ROS under both oxygen-rich and hypoxic conditions. The chemical structure of HB is also more stable than that of HB, since it has the methoxyl group with the electron delocalization across the perylenequinone compound. More importantly, this electron delocalization system is a resonance [81] and not easily degraded through the photochemical reactions in the PDT process. The methoxyl group is hydrophobic and binds to the lipid protein [82], which can improve the permeability of the cell membrane surface to increase the cellular uptake.

However, the methoxyl group of HB makes it less soluble in water, which limits the bioavailability of its function to the effectiveness of PDT. The insoluble property also affects the stability of HB. These are the limitations of HB. Nanotechnology is an option for overcoming these issues. Insoluble property and stability can modify the surface of HB by coating it with hydrophilic, stabilizing, mucoadhesive polymers or copolymers with hydrophilic segments or using surfactants (stabilizer), such as Tween 20, 60, or 80 [83]. The purpose of this is to develop and establish the HB delivery system for targeting cancer cells effectively during PDT efficacy.

Based on the findings, the absorption peak, single oxygen yield, in vitro, in vivo, pharmacokinetics, and toxicity profile of HA and HB are summarized (Table 5).

HA and HB exhibit comparable absorption and emission spectra, pharmacokinetic behavior, phototoxicity profiles, therapeutic selectivity, and consistently high singlet oxygen yields. While HA has been reported to possess slightly higher singlet oxygen quantum yields, HB demonstrates greater versatility in formulation. Encapsulation of HB into liposomal, silica, or graphene oxide nanocarriers enhances its stability and reduces systemic toxicity, making HB more suitable for translational nanoplatform development, NIR-shifted derivatives, and theranostic applications that integrate imaging with therapy. The limitations and inconsistencies of HB and HA are photobleaching susceptibility, as they undergo degradation under prolonged irradiation, lowering PDT efficiency. The absorption, emission, and fluorescence spectra depend on solvent, pH, and aggregation state.

Curcumin [84], berberine [85], hypericin [86], and emodin [87] are the common Traditional Chinese Medicine (TCM) PSs used in PDT. They have different characteristics and properties. “Low solubility” is the most significant problem, other factors that affect the PDT efficacy include photo-stability, tumor selectivity, singlet oxygen yield, and structural modification.

Curcumin has good photostability in dark, but with significant photodegradation rates in both red and blue light at 420 to 430 nm [88]; berberine exhibits limited photostability in aqueous environments due to its aggregation behavior in the blue light at 405 nm [89]; hypericin is photoreactive and its photostability depend on the water-soluble medium in the green-yellow light at 590 nm [90]; emodin is photolabile, unstable and degrades when exposed to light at 200 to 400 nm [91]. 

Curcumin exhibits notable tumor selectivity, accumulating in cancer cells and modulating multiple oncogenic pathways, such as NF-κB [92] and STAT3, PI3K/Akt/mTOR [93], and p53 activation [94]; berberine has the tumor selectivity through different pathways like AMPK activation [95], topoisomerase inhibition [96], NF-κB and STAT3 inhibition [97]; hypericin possesses strong tumor selectivity via preferential accumulation in tumor tissue [98], affinity for tumor-associated proteins and lipids [99], and subcellular localization [100]; emodin has multiple mechanisms, such as immune checkpoint modulation [101], anti-inflammatory effects [102], inhibition of angiogenesis, and metastasis [103] for tumor selectivity.

Curcumin’s singlet oxygen yield is highly dependent on the solvent and environment, which is 0.11 in toluene or acetonitrile but much lower in alcohols or aqueous solutions [104]; the singlet oxygen yield of berberine is also dependent on the solvent and environment, that is, 0.2 to 0.6 in dichloromethane [105]; hypericin has a high singlet oxygen yield of 0.33 in dimethylsufoxide, but it drops significantly in aqueous solutions lead to aggregation [106]; emodin shows a high singlet oxygen yield 0.32 in acetonitrile [107].

The structural modification of curcumin is difficult because of the polyphenolic instability, which can form a negatively charged phenoxide anion, causing rapid degradation [108]; berberine has a rigid isoquinoline framework, and is difficult to make substitution for the charging [109]; the structural modification of hypericin is feasible and it has a polycyclic framework fused with the benzene ring not easily to break [110]; emodin has anthraquinone scaffold and the modification is not easy, because it is planar tricyclic structure [111].

Therefore, HA or HB has more benefits than the above PSs because of the photophysical properties, tumor selectivity, and modifiability (Table 6).

According to the past investigations, there was no large-scale clinical study on HB-PDT or HA-PDT for cancer. It is still in the in vitro or in vivo at this moment. Human trials should start after optimizing the PS delivery, including stability and solubility problems, light dosimetry, and tumor selectivity. In contrast to HA, HB is much valuable to conduct clinical studies, because it is a next-generation PDT agent, which can treat a wide variety of cancers with minimally invasive procedures, and with greater effect than conventional therapy [116].

TCM as a PS in PDT is not a new approach in clinical trials. The most common PS in TCM is “curcumin”. It has been successfully applied to a clinical trial. One of the recent clinical trials entitled “Curcumin vs. Photo-bio-modulation Therapy of Oral Mucositis in Pediatric Patients Undergoing Anti-Cancer Non-invasive Treatment” was completed in 2024 with ClinicalTrials.gov ID NCT06044142 [117]. This was a randomized clinical trial involving 90 patients aged between 3 years and 15 years, which was divided into two groups. Group A submitted to PDT with curcumin activated using blue light sources, such as a laser or LED emitting for approximately 450 nm at 142 J/cm^2^, 100 mW; Group B (control group) submitted to low-level laser therapy in 660 nm with 1 J energy per point at 100 mW power output for 10 s daily. The PDT with curcumin was effective compared to the last therapy in the pediatric patients at different ages.

Curcumin is a suitable benchmark for HB in PDT because of several shared attributes. They are naturally derived photosensitizers with favorable safety profiles, comparable photophysical compatibility for singlet oxygen generation, and established in vitro and in vivo data supporting their therapeutic potential.

Mechanistically, HB and curcumin differ in several key aspects. HB exhibits a broader absorption range peaking in the red region, enabling deeper tissue penetration, whereas curcumin absorbs in the blue-green region, limiting its reach to superficial tissues. HB predominantly generates singlet oxygen via the Type II pathway, while curcumin operates through a mixed Type I/Type II mechanism, resulting in comparatively lower singlet oxygen yield. Subcellular localization also diverges: HB accumulates in mitochondria and lysosomes, initiating caspase-dependent mitochondrial apoptosis, whereas curcumin localizes in the cytosol and nucleus, exerting multifactorial effects through ROS-mediated signaling, including NF-κB and MAPK pathways. These mechanistic distinctions underscore HB’s enhanced photodynamic specificity and deeper therapeutic reach relative to curcumin.

In addition, there have been two nanoformulations of curcumin PDT clinical studies, which are “Study Investigating the Ability of Plant Exosomes to Deliver Curcumin to Normal and Colon Cancer Tissue” (NCT01294072) and “Prophylactic Topical Agents in Reducing Radiation-Induced Dermatitis in Patients With Non-inflammatory Breast Cancer (Curcumin-II)” (NCT02556632). The first clinical study investigates the effect of exosomally delivered curcumin on the immune modulation, cellular metabolism, and phospholipid profile of normal and malignant colon cells in three groups who are undergoing surgery for newly diagnosed colon cancer. Group 1 is curcumin alone, group 2 is curcumin with plant exosomes, and group 3 is the control without treatment. This project is ongoing [118]. Another clinical study was completed in 2016 and investigated the effectiveness of Curcumin gel (curcumin-based gel) or HPR Plus™ in reducing radiation dermatitis in three groups of breast cancer patients. Group 1 was curcumin-based gel, group 2 was HPR Plus, and group 2 was the control (placebo gel). The results indicated that the prophylactic topical agents, such as curcumin-based gel or HPR Plus, may reduce the severity of the radiation-induced dermatitis by minimizing water loss and inflammation during radiation therapy [119].

In fact, curcumin and HB are natural PSs; the clinical stage and regulatory status are different to the synthetic PSs (Table 7).

Thus, HB may be a potential candidate used as PS in PDT, but it must follow the following criteria before clinical study, which are “quality”, “safety”, “efficacy”, “pharmacokinetics”, and “regulatory compliance” [126]. To fulfill the above requirements, the preclinical evaluation should include in vitro and in vivo studies, toxicology, and the mechanism of action [127]. It can be designed as a clinical trial after finishing these conditions, which consists of three phases. Each phase corresponds to the patient’s health. Phase I is the safety and dosage of the PS; Phase II is the efficacy of the treatment; Phase III is the comparison to the other therapy.

## 7. Conclusions

Hypocrellin has a photodynamic action on cancers. HB is a much better PS than HA. However, much more work needs to be conducted, especially regarding the solubility and stability of HB, as well as in enhancing singlet oxygen yield, tissue penetration, and combinational PDT with immunotherapy or chemotherapy through the nanoformulations. It should also be compared to other TCM PSs, such as curcumin, berberine, hypericin, and emodin. In addition, a large-scale clinical trial of HB-PDT should be conducted after fulfilling the requirements of “quality”, “safety”, “efficacy”, “pharmacokinetics”, and “regulatory compliance” through the incorporation with nanotechnology. “Quality” of the hypocrellin depends on optimizing its formulation, protecting it from photodegradation, and enhancing its solubility and delivery. “Safety” is in reference to light exposure to minimize off-target effects and phototoxicity during the PDT process. “Efficacy” refers to the correct targeting of cancer. “Pharmacokinetics” is in reference to the dose usage for toxicity and the mechanism of action. “Regulatory compliance” refers to the international regulatory frameworks for the treatment, such as those from the FDA, through the implementation of Good Manufacturing Practices (GMP).

## 8. Future Aspects

Recent advances in the PDT of HA and HB against cancer have focused on optimizing their physicochemical properties and therapeutic efficacy through nanotechnology, targeted delivery, and photophysical enhancement. Nanoformulation strategies have significantly improved the solubility, photostability, and bioavailability of both HA and HB, mitigating their inherent limitations such as poor aqueous solubility and aggregation. In particular, HB-loaded nanocarriers have demonstrated enhanced tumor selectivity and controlled release profiles, establishing a robust delivery system for cancer targeting. Moreover, HB-PDT is increasingly being explored in combination with other therapeutic modalities—including chemotherapy, immunotherapy, and radiotherapy—to achieve synergistic effects. These multimodal approaches not only amplify antitumor efficacy but also help overcome drug resistance and reduce the required doses of individual therapies, thereby minimizing long-term side effects [128,129,130,131]. Such integrative strategies may compensate for or eliminate the limitations of HB-PDT, positioning it as a versatile and adaptable platform for precision oncology.

## Figures and Tables

**Table 3:** Examples of the traditional strategy of HA-PDT for cancer.

	Study	Experiment Parameters	Consequence	References
1	Hypocrellin A-based photodynamic action induces apoptosis in A549 cells through ROS-mediated mitochondrial signaling pathway	Absorption (λ_abs_): 540 to 560 nm (dominant band), secondary peak at 465 nm. Absorption maximum (λ_abs max_): 550 nm for molar extinction coefficients (ε): ~3.1 × 10^4^ M^−1^·cm^−1^ Emission (λ_em_): 630 to 660 nm with λ_em max_: 645 nm Singlet oxygen quantum yields (ΦΔ): ~0.63 Cytotoxicity IC_50_: ~0.8 to 1.2 μM in A549 cells	HA-PDT was associated with cell shrinkage, externalization of cell membrane phosphatidylserine, DNA fragmentation, and mitochondrial disruption, as well as pronounced release of cytochrome c, and activation of caspase-3, -9, and -7.	[72]
2	Photodynamic effects of hypocrellin A on three human malignant cell lines by inducing apoptotic cell death	Absorption (λ_abs_): 540 to 560 nm (dominant band), secondary peak at 465 nm. Absorption maximum (λ_abs max_): 550 nm for molar extinction coefficients (ε): ~3.0 × 10^4^ M^−1^·cm^−1^ Emission (λ_em_): 630 to 660 nm with λ_em max_: 645 nm Singlet oxygen quantum yields (ΦΔ): ~0.63 Cytotoxicity IC_50_: ~0.8 to 1.5 μM in HeLa, MGC-803, and HIC cells	HA-PDT induced apoptosis or necrosis, evidenced by morphological changes, DNA fragmentation, and a decrease in mitochondrial dehydrogenase activity in human malignant epithelioid cells.	[73]
3	Toxicity and phototoxicity of hypocrellin A on malignant human cell lines, evidence of a synergistic action of photodynamic therapy with Imatinib mesylate	Absorption (λ_abs_): 540 to 560 nm (dominant band), secondary peak at 465 nm. Absorption maximum (λ_abs max_): 550 nm for molar extinction coefficients (ε): ~3.0 × 10^4^ M^−1^·cm^−1^ Emission (λ_em_): 630 to 660 nm with λ_em max_: 645 nm Singlet oxygen quantum yields (ΦΔ): ~0.63 Cytotoxicity IC_50_: ~0.6 to 1.5 μM in HeLa, Calu, and K562 cell lines	The phototoxicity of HA in epithelial cell lines demonstrated a synergy between imatinib mesylate and photodynamic therapy to circumvent imatinib mesylate resistance.	[74]

**Table 4:** Examples of the nanotechnology of HA-PDT for cancer.

	Study	Experiment Parameters	Consequence	References
1	Enhancing the photosensitivity of hypocrellin A by perylene diimide metallacage-based host-guest complexation for photodynamic therapy	Absorption (λ_abs_): 540 to 560 nm (dominant band), secondary peak at 465 nm. Absorption maximum (λ_abs max_): 550 nm for molar extinction coefficients (ε): ~3.0 to 3.6 × 10^4^ M^−1^·cm^−1^ Emission (λ_em_): 630 to 660 nm with λ_em max_: 645 nm Singlet oxygen quantum yields (ΦΔ): ~0.63 to 0.72 Cytotoxicity IC_50_: ~0.5 to 1.2 μM in HeLa, MCF-7 cells In vivo pharmacokinetics: reduce systemic toxicity	HA perylene diimide-based metallacages displayed excellent anticancer activities upon light irradiation in PDT and enhanced the photosensitivity of conventional photosensitizers via host-guest complexation-based fluorescence resonance energy transfer.	[75]
2	A new near-infrared photosensitizing nanoplatform containing blue-emitting up-conversion nanoparticles and hypocrellin A for photodynamic therapy of cancer cells	Absorption (λ_abs_): 540 to 560 nm (dominant band), secondary peak at 465 nm. Absorption maximum (λ_abs max_): 550 nm for molar extinction coefficients (ε): ~3.0 × 10^4^ M^−1^·cm^−1^ Emission (λ_em_): 630 to 660 nm with λ_em max_: 645 nm Singlet oxygen quantum yields (ΦΔ): ~0.65 Cytotoxicity IC_50_: ~0.6 to 1.0 μM in HeLa, and MCF-7 cells	Tween 20-up-conversion nanoparticles@HA complexes-PDT efficiently produced singlet oxygen to kill cancer cells, exhibited positive contrast effects on the magnetic resonance imaging (MRI) and computed tomography (CT) imaging.	[76]
3	Transferrin-modified nanoparticles for photodynamic therapy enhance the antitumor efficacy of Hypocrellin A	Absorption (λ_abs_): 540 to 560 nm (dominant band), secondary peak at 465 nm. Absorption maximum (λ_abs max_): 550 nm for molar extinction coefficients (ε): ~3.0 × 10^4^ M^−1^·cm^−1^ Emission (λ_em_): 630 to 660 nm with λ_em max_: 645 nm Singlet oxygen quantum yields (ΦΔ): ~0.63 Cytotoxicity IC_50_: ~0.6 to 0.9 μM in HeLa, and HepG2 cells	Poly(D, L-Lactide-co-glycolide) and carboxymethyl chitosan nanoparticle-loaded with HA enhanced PDT therapeutic efficacy, which caused cell apoptosis in tumor tissue and slight side effects in normal organs.	[77]
4	Hypocrellin A-cisplatin-intercalated hectorite nano formulation for chemo-photodynamic tumor-targeted synergistic therapy	Absorption (λ_abs_): 540 to 560 nm (dominant band), secondary peak at 465 nm. Absorption maximum (λ_abs max_): 550 nm for molar extinction coefficients (ε): ~3.0 × 10^4^ M^−1^·cm^−1^ Emission (λ_em_): 630 to 660 nm with λ_em max_: 645 nm Singlet oxygen quantum yields (ΦΔ): ~0.62 Cytotoxicity IC_50_: ~0.5 to 0.8 μM in synergistic therapy with cisplatin	HA-cisplatin-intercalated hectorite nano formulation-PDT possessed stable light absorption, high oxygen generation with controlled drug release efficacy to induce apoptosis and necrosis for targeted and effective esophageal cancer treatment.	[78]

**Table 5:** A comparative study of HA and HB in PDT.

	Hypocrellin B (HB)	Hypocrellin A (HA)
**Absorption and emission peaks**	Absorption (λ_abs_): 540 to 560 nm (dominant band), secondary peak at 465 nm, Absorption maximum (λ_abs max_): 550 nm; Emission (λ_em_): 630 to 660 nm with λ_em max_: 645 nm	Absorption (λ_abs_): 540 to 560 nm (dominant band), secondary peak at 465 nm, Absorption maximum (λ_abs max_): 550 nm; Emission (λ_em_): 630 to 660 nm with λ_em max_: 645 nm
**Single oxygen yield**	High (0.50 or above, depend on the solvent)	High (0.60 or above, depend on the solvent)
**In vitro studies**	Investigate different cancer cell lines (Table 1)	Slightly limited than HB (Table 3)
**In vivo studies**	Mice models	Slightly limited than HB
**Pharmacokinetics**	Selective target and uptake for cancer	Similarly to HB
**Toxicity profile**	Low dark toxicity	Low dark toxicity

**Table 6:** Benefits of using HA or HB over the other TCM PSs.

PS	Source	Photo-Stability	Tumor Selectivity	Singlet Oxygen Yield	Structural Modification
Hypocrellin (HA and HB)	Fungus *Hypocrella bambusae*	Excellent	Excellent (After chemical and structural modification)	High	High
Curcumin	*Curcuma longa* [112]	Poor	Satisfaction	Low	Limit
Berberine	*Coptidis rhizome* [113]	Satisfaction	Satisfaction	Average	Limit
Hypericin	St. John’s wort [114]	Good	Good	High	Satisfaction
Emodin	Rhubarb [115]	Satisfaction	Satisfaction	High	Satisfaction

**Table 7:** Clinical stage and regulatory status of synthetic vs. natural PSs.

	Synthetic PSs (Photofrin II, Methyl Aminolevulinate)	Natural PSs (Curcumin, HB)
**Clinical stage**	Completed the multiple indications for cancers [120,121]	Curcumin: clinical phase II [122] HB: Not in clinical phase and still in pre-clinical, in vitro, and in vivo studies
**Regulatory status**	Approved by the Food and Drug Administration (FDA) [123,124]	Curcumin and HB are not FDA-approved [55,125]

## Data Availability

No new data were created or analyzed in this study. Data sharing is not applicable.

## Abbreviations

ALA	5-aminolevulinic acid
AMPK	AMP-activated protein kinase
Bcl-2	B-cell lymphoma 2
Bcl-xL	B-cell lymphoma-extra large
CNKI	China National Knowledge Infrastructure
FDA	Food and Drug Administration
HA	Hypocrellin A
HB	Hypocrellin B
IARC	International Agency for Research on Cancer
LED	Light Emitting Diode
MAL	Methyl aminilevulinate
NF-κB	Nuclear Factor kappa-light-chain-enhancer of activated B cells
p53	Tumor Protein p53
PDT	Photodynamic Therapy
PEG-PLA	Polyethylene glycol-polylactic acid
PI3K/Akt/mTOR	Phosphoinositide 3-kinase/Akt/Mammalian target of rapamycin
PLGA	Poly(lactic-co-glycolic acid)
PS	Photosensitizer
ROS	Reactive Oxygen Species
STAT3	Signal Transducer and Activator of Transcription 3
TCM	Traditional Chinese Medicine
TNF-α	Tumor Necrosis Factor-alpha
